# Traumatic injuries among adult obese patients in southern Taiwan: a cross-sectional study based on a trauma registry system

**DOI:** 10.1186/s12889-016-2950-z

**Published:** 2016-03-18

**Authors:** Jung-Fang Chuang, Cheng-Shyuan Rau, Pao-Jen Kuo, Yi-Chun Chen, Shiun-Yuan Hsu, Hsiao-Yun Hsieh, Ching-Hua Hsieh

**Affiliations:** Department of Trauma Surgery, Kaohsiung Chang Gung Memorial Hospital and Chang Gung University College of Medicine, No.123, Ta-Pei Road, Niao-Song District, Kaohsiung City, 833 Taiwan; Department of Neurosurgery, Kaohsiung Chang Gung Memorial Hospital and Chang Gung University College of Medicine, Kaohsiung City, Taiwan; Department of Plastic and Reconstructive Surgery, Kaohsiung Chang Gung Memorial Hospital and Chang Gung University College of Medicine, Kaohsiung City, Taiwan

**Keywords:** Trauma, Obese, Injury Severity Score, Fracture, Mortality, Hospital length of stay

## Abstract

**Background:**

The adverse impact of obesity has been extensively studied in the general population; however, the added risk of obesity on trauma-related mortality remains controversial. This study investigated and compared mortality as well injury patterns and length of stay (LOS) in obese and normal-weight patients hospitalized for trauma in the hospital and intensive care unit (ICU) of a Level I trauma center in southern Taiwan.

**Methods:**

Detailed data of 880 obese adult patients with body mass index (BMI) ≥ 30 kg/m^2^ and 5391 normal-weight adult patients (25 > BMI ≥ 18.5 kg/m^2^) who had sustained a trauma injury between January 1, 2009 and December 31, 2013were retrieved from the Trauma Registry System. Pearson’s chi-squared, Fisher’s exact, and independent Student’s t-tests were used to compare differences between groups. Propensity score matching with logistic regression was used to evaluate the effect of obesity on mortality.

**Results:**

In this study, obese patients were more often men, motorcycle riders and pedestrians, and had a lower proportion of alcohol intoxication compared to normal-weight patients. Analysis of Abbreviated Injury Scale scores revealed that obese trauma patients presented with a higher rate of injury to the thorax, but a lower rate of facial injuries than normal-weight patients. No significant differences were found between obese and normal-weight patients regarding Injury Severity Score (ISS), Trauma-Injury Severity Score (TRISS), mortality, the proportion of patients admitted to the ICU, or LOS in ICU. After propensity score matching, logistic regression of 66 well-matched pairs did not show a significant influence of obesity on mortality (odds ratio: 1.51, 95 % confidence interval: 0.54–4.23 *p* = 0.438). However, significantly longer hospital LOS (10.6 vs. 9.5 days, respectively, *p* = 0.044) was observed in obese patients than in normal-weight patients, particularly obese patients with pelvic, tibial, or fibular fractures.

**Conclusion:**

Compared to normal-weight patients, obese patients presented with different injury characteristics and bodily injury patterns but no difference in mortality.

**Electronic supplementary material:**

The online version of this article (doi:10.1186/s12889-016-2950-z) contains supplementary material, which is available to authorized users.

## Background

The rising prevalence of obesity has been described as a global pandemic [1, 2]. According to data from the United States National Health and Nutrition Examination Survey, more than one-third of adults were obese in 2011-2012 [3]. In Taiwan, the prevalence of overweight and obesity among adults increased from 33 to 44 % according to the results of the Nutrition and Health Survey conducted by the Taiwanese government between 1993 and 1996 and between and 2008 [4]. It is projected that by 2020, three in four Americans and seven of ten British citizens will be overweight [5]. Elevated body mass index (BMI) had been suggested to intensify the energy dissipated in a crash and therefore potentially increase vulnerability to serious injury or death [6]. Some investigations have reported that trauma induces excessive persistent inflammatory response, which could amplify the adverse effects of critical illness in obese patients [7, 8]. Likewise, obesity in trauma patients is reportedly associated with increased complications and worse outcomes [9–11]. Obese trauma patients are more likely to require mechanical ventilation, develop multiple organ failure, and spend more time in intensive care units (ICUs) [12, 13].

The most researched outcome related to trauma patients is mortality, which is the most serious outcome on the spectrum. However, the evidence for the relationship between trauma-related mortality and obesity is conflicting [14]. Several studies have reported an association between obesity and mortality [7, 10, 15, 16], and meta-analyses that pool multiple studies support the hypothesis that obesity results in higher mortality among patients with traumatic injuries; however, these associations are much less profound [9], and several other studies found no difference in mortality [17–22].

Increased understanding of the epidemiology of trauma is vital in order for local trauma systems to cope with a rising number of obese patients. In Taiwan, the mechanism of traumatic injury differs from that of Western countries, with motorcycle accidents comprising the majority of trauma injuries that require admission to the hospital, followed by falls and motor vehicle accidents [23–25]. Although almost all motorcycles are forbidden on highways in Taiwan and most traffic accidents occur in relatively crowded streets at relatively low velocities [23], a previous study indicated that motorcyclist fatalities still account for nearly 60 % of all driving fatalities in Taiwan [26]. Because the mechanism of traumatic injury in Taiwan is distinct from that in many Western countries, the main aim of this study was to investigate and compare mortality rates as well injury patterns and length of stay (LOS) in hospital or intensive care units (ICUs) between obese and normal-weight patients hospitalized for treatment of traumatic injuries in a Level I trauma center in southern Taiwan.

## Methods

### Ethics statement

This study was pre-approved by the Institutional Review Board (IRB) of the Chang Gung Memorial Hospital (approval number 103-7105B). Informed consent was waived according to IRB regulations.

### Study design

This retrospective study reviewed all data added to the Trauma Registry System from January 1, 2009 to December 31, 2013 in a 2400-bed facility and Level I regional trauma center that provides care to trauma patients primarily from southern Taiwan. The inclusion criteria were (1) obese patients with BMI ≥ 30 kg/m^2^ and normal-weight patients with BMI < 25 but ≥ 18.5 kg/m^2^ according definitions from the World Health Organization [27, 28], and (2) hospitalization for treatment of traumatic injury. To compare injury patterns, mechanisms, severity, and mortality of obese patients to those of normal-weight patients hospitalized for trauma, we reviewed all 16,548 hospitalized and registered patients added to the Trauma Registry System from January 1, 2009 to December 31, 2013. Detailed patient information was retrieved from the Trauma Registry System of our institution, including data regarding age, sex, admission vital signs, transportation, injury mechanism, initial Glasgow Coma Scale (GCS) in the emergency department, Abbreviated Injury Scale (AIS) severity score for each body region, Injury Severity Score (ISS), New ISS (NISS), Trauma-ISS (TRISS), hospital length of stay (LOS), LOS in ICU, in-hospital mortality, and rates of associated complications. Pre-existing comorbidities and chronic diseases including diabetes mellitus (DM), hypertension (HTN), coronary artery diseases (CAD), congestive heart failure (CHF), cerebrovascular accident (CVA), and end-stage renal disease (ESRD) were also identified. Methods of transportation included patient transfer to hospital by fire-based emergency medical services (EMS), private vehicle (including friends, relatives, bystanders, and patients driving themselves), or transfer from other hospitals or clinics by hospital-based ambulance. In this study, the primary outcome was the injury severity according to different scoring system (GCS, AIS, ISS, NISS, TRISS), while the secondary outcome was LOS, LOS in ICU, and in-hospital mortality. Blood alcohol concentration (BAC) of 50 mg/dL at the time of arrival to the hospital was defined as a cut-off values and the legal limit for drivers in Taiwan. Odd ratios (ORs) of the associated conditions and injuries of obese and normal-weight patients involved in motorcycle accidents were calculated with 95 % confidence intervals (CIs). The data collected regarding the combined population of drivers and pillions (riders) were compared using IBM SPSS Statistics for Windows, version 20.0 (IBM Corp., Armonk, NY, USA) to perform Pearson’s chi-squared, Fisher’s exact, or independent Student’s t-tests, as applicable. To minimize confounding effects due to nonrandomized assignment in the assessment of mortality, propensity scores were calculated using a logistic regression model and the following covariates: age; HTN; alcohol intoxication (BAC > 50 mg/dL); GCS; injuries to the head/neck, thorax, or extremities based on AIS; and ISS. A 1:1 matched study group was created by the Greedy method with NCSS software (NCSS 10, NCSS Statistical software, Kaysville, Utah). After amending these confounding factors, binary logistic regression was used in the evaluation of interventional factor of obesity on mortality. All results are presented as means ± standard errors. *P*-values less than 0.05 were considered statistically significant.

## Results

### Injury characteristics of obese patients

Among 9553 adult patients, 880 (9.2 %) and 5391(56.4 %) were obese and normal weight, respectively (Table 1). Among these, we found were significantly more obese men and normal-weight women. The mean ages of the obese and normal-weight patients were 42.8 ± 13.4 and 42.9 ± 13.8 years, respectively (Table 1). Significantly higher incidence rates of pre-existing comorbidities and chronic diseases including DM (OR 2.5, 95 % CI 1.99–3.03; *p* < 0.001), HTN (OR 3.1, 95 % CI 2.62–3.67; *p* < 0.001), and CHF (OR 3.8, 95 % CI 1.57–9.18; *p* = 0.005) were found among obese patients than among normal-weight patients. No differences in transportation methods were found between obese and normal-weight patients. Among obese adult patients, motorcycle drivers were most commonly admitted (51.4 %), followed by falls (17.6 %) and unspecified traumatic injuries (14.9 %). There were no significant differences between groups in the time of arrival at the emergency department. Positive BAC was less frequent among obese patients than among normal-weight patients (7.0 vs. 9.3 %, respectively; *p* = 0.032).

**Table 1:** Demographics and injury characteristics of obese and normal-weight adult trauma patients

Variables	Obese BMI ≥ 30 *n* = 880	Normal 25 > BMI ≥ 18.5 *n* = 5391	*Odds ratio* *(95 %)*	*P*
Gender				
Male	599(68.1)	3279(60.8)	1.4(1.18–1.60)	<0.001
Female	281(31.9)	2112(39.2)	0.7(0.63–0.85)	<0.001
Age	42.8 ± 13.4	42.9 ± 13.8	—	0.907
Comorbidity				
DM	136(15.5)	374(6.9)	2.5(1.99–3.03)	<0.001
HTN	249(28.3)	609(11.3)	3.1(2.62–3.67)	<0.001
CAD	13(1.5)	46(0.9)	1.7(0.94–3.24)	0.075
CHF	8(0.9)	13(0.2)	3.8(1.57–9.18)	0.005
CVA	15(1.7)	67(1.2)	1.4(0.78–2.42)	0.264
ESRD	0(0.0)	5(0.1)	—	1.000
Transpose, n(%)				
EMS	341(38.8)	2007(37.2)	1.1(0.92–1.24)	0.387
Private vehicle	240(27.3)	1628(30.2)	0.9(0.74–1.02)	0.078
Transferred	299(34.0)	1756(32.6)	1.1(0.92–1.24)	0.411
Mechanism, n(%)				
Motor vehicle driver	23(2.6)	98(1.8)	1.5(0.92–2.30)	0.112
Motor vehicle passenger	13(1.5)	51(0.9)	1.6(0.85–2.90)	0.146
Motorcycle driver	452(51.4)	2559(47.5)	1.2(1.01–1.35)	0.032
Motorcycle pillion	14(1.6)	142(2.6)	0.6(0.34–1.04)	0.065
Bicycle	19(2.2)	166(3.1)	0.7(0.43–1.12)	0.135
Pedestrian	6(0.7)	94(1.7)	0.4(0.17–0.89)	0.020
Fall	155(17.6)	1061(19.7)	0.9(0.72–1.05)	0.150
Penetrating injury	39(4.4)	330(6.1)	0.7(0.51–1.00)	0.053
Burn	28(3.2)	155(2.9)	1.1(0.74–1.67)	0.616
Struck by/against	131(14.9)	735(13.6)	1.1(0.91–1.36)	0.318
Alcohol > 50, n(%)	62(7.0)	500(9.3)	0.7(0.56–0.98)	0.032
Time, n(%)				
7:00–17:00	515(58.5)	2964(55.0)	1.2(1.00–1.34)	0.053
17:00–23:00	228(25.9)	1529(28.4)	0.9(0.75–1.04)	0.133
23:00–7:00	137(15.6)	898(16.7)	0.9(0.76–1.12)	0.420
GCS	14.5 ± 2.0	14.3 ± 2.2	—	0.041
≤ 8	27(3.1)	258(4.8)	0.6(0.42–0.94)	0.023
9–12	38(4.3)	192(3.6)	1.2(0.86–1.74)	0.268
≥ 13	815(92.6)	4941(91.7)	1.1(0.87–1.50)	0.336
AIS, n(%)				
Head/Neck	195(22.2)	1349(25.0)	0.9(0.72–1.01)	0.067
Face	137(15.6)	1055(19.6)	0.8(0.62–0.92)	0.005
Thorax	130(14.8)	625(11.6)	1.3(1.08–1.62)	0.007
Abdomen	64(7.3)	356(6.6)	1.1(0.84–1.46)	0.462
Extremity	659(74.9)	3952(73.3)	1.1(0.92–1.28)	0.325
ISS	8.2 ± 7.3	8.2 ± 7.2	—	0.790
< 16	754(85.7)	4621(85.7)	1.0(0.81–1.22)	0.978
16–24	82(9.3)	541(10.0)	0.9(0.72–1.18)	0.510
≥ 25	44(5.0)	229(4.2)	1.2(0.85–1.65)	0.311
NISS	9.6 ± 8.7	9.5 ± 8.8	—	0.712
TRISS	0.971 ± 0.093	0.968 ± 0.102	—	0.347
Mortality, n(%)	11(1.3)	63(1.2)	1.1(0.56–2.04)	0.836
LOS (days)	10.6 ± 11.3	9.2 ± 10.0	—	<0.001
ICU				
Patients, n(%)	149(16.9)	884(16.4)	1.0(0.86–1.26)	0.692
< 16	63(7.2)	353(6.5)	1.1(0.83–1.45)	0.499
16–24	49(5.6)	335(6.2)	0.9(0.65–1.21)	0.459
≥ 25	37(4.2)	196(3.6)	1.2(0.81–1.67)	0.408
LOS in ICU (days)	9.2 ± 9.7	8.8 ± 11.0	—	0.660
< 16	7.5 ± 6.3	8.7 ± 9.6	—	0.355
16–24	7.7 ± 8.6	7.0 ± 10.6	—	0.689
≥ 25	14.2 ± 13.7	12.1 ± 13.2	—	0.371

### Injury severity among obese patients

As shown in Table 1, GCS scores were significantly higher in obese patients than in normal-weight patients (14.5 ± 2.0 vs. 14.3 ± 2.2, respectively, *p* = 0.041). However, the difference was less than 1 point. The most common GCS among patients in both groups (obese and normal-weight adults) were ≥13. However, fewer obese adult patients had a GCS ≤ 8 compared to those of normal-weight patients (3.1 vs. 4.8 %, respectively, *p* = 0.023). Analysis of AIS revealed that obese patients had sustained significantly higher rates of thoracic injury (14.8 vs. 11.6 %, respectively, *p* = 0.007) than normal-weight patients, while normal-weight patients had sustained significantly higher rates of facial injury (19.6 vs. 15.6 %, respectively, *p* = 0.005). No significant differences were found between obese and normal-weight adult patients regarding ISS (regardless of injury severity subgroup), NISS, TRISS, and in-hospital mortality. After propensity score matching, 66 well-balanced pairs of patients were used for outcome comparison. In these propensity score-matched patients, there was no significant difference in age; HTN; alcohol intoxication (BAC > 50 mg/dL); GCS; injury to head/neck, thorax, or extremities based on AIS; and ISS (Table 2). In addition, logistic regression analysis did not show that obesity significantly influenced mortality (OR: 1.51, 95 % CI: 0.54–4.23 *p* = 0.438). However, obese patients had significantly longer hospital LOS than normal-weight adult patients (10.6 vs. 9.2 days, respectively, *p* < 0.001). No significant differences were found regarding the proportion of patients admitted to the ICU (16.9 vs. 16.4 %, respectively, *p* = 0.692) or LOS in the ICU (9.2 vs. 8.8 days, respectively, *p* = 0.660), regardless of injury severity.

**Table 2:** Significant covariates of obese and normal-weight patients before and after propensity score matching (1:1 matching)

	Before matching				After matching			
Variables	Death *n* = 74	Survival *n* = 6197	*Odds ratio * *(95 %)*	*P*	Death *n* = 66	Survival *n* = 66	*Odds ratio * *(95 %)*	*P*
Age	50.0 ± 12.5	42.8 ± 13.7	—	<0.001	49.9 ± 12.7	48.7 ± 12.7	—	0.509
HTN	16(21.6)	842(13.6)	1.8(1.00–3.07)	0.046	15(22.7)	15(22.7)	1.0(0.44–2.26)	
Alcohol > 50, n(%)	15(20.3)	547(8.8)	2.6(1.48–4.66)	0.001	12(18.2)	12(18.2)	1.0(0.41–2.42)	
GCS	6.3 ± 4.5	14.4 ± 2.0	—	<0.001	6.7 ± 4.6	7.4 ± 4.6	—	0.257
AIS, n(%)								
Head/Neck	65(87.8)	1479(23.9)	23.0(11.45–46.37)	<0.001	58(87.9)	58(87.9)	1.0(0.35–2.84)	
Thorax	15(20.3)	740(11.9)	1.9(1.06–3.32)	0.029	13(19.7)	13(19.7)	1.0(0.42–2.36)	
Extremity	21(28.4)	4590(74.1)	0.1(0.08–0.23)	<0.001	19(28.8)	19(28.8)	1.0(0.47–2.13)	
ISS	30.6 ± 17.9	7.9 ± 6.5	—	<0.001	25.7 ± 11.0	23.8 ± 10.5	—	0.124

### Physiological response and procedures performed in the emergency room

Obese patients exhibited higher ORs for presenting to the emergency room with worse measures of heart rate (OR 1.5, 95 % CI 1.25–1.75; *p* < 0.001) than normal-weight patients (Table 3). There were no significant differences in other measures, including GCS < 13, systolic blood pressure (SBP) < 90 mmHg, or respiratory rate < 10 or > 29. In addition, obese adult patients had lower odds of requiring intubation in the emergency department (OR 0.5, 95 % CI 0.27–0.98; *p* = 0.041), but there were no significant differences in cardiopulmonary resuscitation, chest tube insertion, and blood transfusion between obese and normal-weight patients.

**Table 3:** Physiological response and procedures performed upon arrival at the emergency department

Variables	Obese BMI ≥ 30 *n* = 880	Normal 25 > BMI ≥ 18.5 *n* = 5391	*Odds ratio* *(95 %)*	*P*
Physiology at ER, n(%)				
GCS < 13	65(7.4)	450(8.3)	0.9(0.67–1.15)	0.336
SBP < 90 mmHg	22(2.5)	122(2.3)	1.1(0.70–1.75)	0.663
Heart rate > 100 beats/min	221(25.1)	997(18.5)	1.5(1.25–1.75)	<0.001
Respiratory rate < 10 or >29	7(0.8)	25(0.5)	1.7(0.74–3.99)	0.201
Procedures at ER, n(%)				
Cardiopulmonary resuscitation	1(0.1)	8(0.1)	0.8(0.10–6.13)	1.000
Intubation	10(1.1)	118(2.2)	0.5(0.27–0.98)	0.041
Chest tube	9(1.0)	79(1.5)	0.7(0.35–1.39)	0.301
Blood transfusion	27(3.1)	128(2.4)	1.3(0.85–1.98)	0.219

### Associated injuries among obese patients

Additional file 1: Table S1 shows the incidence of associated injuries in obese and normal-weight adult patients. A significantly lower percentage of obese patients had sustained cranial, maxillary, mandibular, pneumothorax, and clavicle fractures (Table 4). In contrast, a significantly higher percentage of obese patients had sustained humeral fractures (OR = 2.1, 95 % CI = 1.62–2.82; *p* < 0.001). Additional file 1: Table S2 shows the LOS in hospital for obese and normal-weight patients. As shown in Table 5, the LOS in hospital was significantly longer in obese patients with pelvic (25.8 vs. 17.0 days, respectively, *p* = 0.010), tibial (19.8 vs. 13.6 days, respectively, *p* = 0.002), and fibular fractures (18.3 vs. 13.6 days, respectively, *p* = 0.027), than in normal-weight patients. However, the LOS in hospital was significantly shorter in obese patients with cerebral contusions and orbital fractures.

**Table 4:** Significant associated injuries among obese and normal-weight patients

Variables	Obese BMI ≥ 30 *n* = 880, n(%)	Normal 25 > BMI ≥ 18.5 *n* = 5391, n(%)	*Odds ratio* *(95 %)*	*P*
Cranial fracture	38(4.3)	339(6.3)	0.7(0.48–0.95)	0.023
Maxillary fracture	36(4.1)	407(7.5)	0.5(0.37–0.74)	<0.001
Mandibular fracture	14(1.6)	151(2.8)	0.6(0.32–0.98)	0.038
Pneumothorax	7(0.8)	99(1.8)	0.4(0.20–0.93)	0.026
Clavicle fracture	57(6.5)	519(9.6)	0.7(0.49–0.86)	0.003
Humeral fracture	72(8.2)	216(4.0)	2.1(1.62–2.82)	<0.001

**Table 5:** Significant differences in length of stay (LOS) in the hospital and associated injuries among obese and normal-weight patients

Variables	Obese BMI ≥ 30 *n* = 880	Normal 25 > BMI ≥ 18.5 *n* = 5391	*P*
LOS (days)			
Cerebral contusion	0.8 ± 8.7	16.5 ± 14.9	0.002
Orbital fracture	7.4 ± 6.2	11.7 ± 7.9	0.033
Pelvic fracture	25.8 ± 18.1	17.0 ± 12.7	0.010
Tibial fracture	19.8 ± 16.4	13.6 ± 12.1	0.002
Fibular fracture	18.3 ± 12.9	13.6 ± 11.9	0.027

## Discussion

This study compared the demographics and characteristics of injuries observed in a population of obese adult patients to those of normal-weight patients hospitalized at a Level I trauma center. The results of this study indicate that obese patients were more often men, more often motorcycle riders and pedestrians, and were less often positive for alcohol intoxication than normal-weight patients. Compared to normal-weight patients, obese patients presented with different bodily injury patterns and had longer hospital stays, particularly for those obese patients with pelvic, tibial, and fibular fractures. Importantly, logistic regression analysis of well-matched pairs after propensity score matching did not show a significant influence of obesity on mortality.

In this study, positive BAC was less frequently observed among obese patients (7.0 vs. 9.3 %, respectively; *p* = 0.032). In the United States, approximately one-third of adult men and women had the co-occurrence of obesity and alcohol use [29]. Unsurprisingly, the prevalence of this high-risk behavior of excessive alcohol use may also contribute to excess body weight [29]. It is unknown whether the reduced frequency of alcohol intoxication among the obese patients in this study was due to their behavior at the time of injury or because more alcohol is necessary to reach the same BAC in a heavier body [30]. This study also revealed that obese adult patients had lower odds of requiring intubation in the emergency department. Notably, obese patients have anatomic and physiologic characteristics that make their intubation more challenging; however, in a study of 1435 intubated patients evaluated during a 3-year study period, BMI was not an independent risk factor for failed intubations (in the field or in emergency departments), post-intubation airway complications, or death [31].

Obesity has significant implications for the patterns of injury. Some authors have reported that obese trauma patients sustain more pelvic, rib, and lower extremity fractures, but fewer liver injuries, mandibular fractures, and cerebral injuries [18]. Other studies have demonstrated a similar pattern of fewer head injuries, but more chest and lower extremity injuries [17]. Some studies have demonstrated that obese women are at increased risk of ankle, leg, humerus, and vertebral column fractures and a lower risk of wrist, hip, and pelvis fractures compared to non-obese women [32]; however, multiple rib fractures are associated with obesity in men [32]. In this study, obese patients presented with facial injuries at a lower rate, but presented with a higher rate of injury to the thorax compared to rates in normal-weight patients. Obese patients also had different associated injuries, including fewer cranial, maxillary, mandibular, clavicle, and pneumothorax fractures, as well as more humoral fractures, than normal-weight patients. The results of this study are similar to those of a prior study that demonstrated no difference between obese and lean patients in the type of traumatic brain injury, including cerebral contusion, epidural hematoma (EDH), subarachnoid hemorrhage (SAH), and subdural hematoma (SDH) in a retrospective review of all blunt trauma patients admitted to the ICU at a Level I trauma center [33]. Although increased chances of maximum head injury (AIS = 6) among obese patients (RR 1.97; 95 % CI: 1.52–2.55; *p* = 0.003) were reported in a study of only drivers or passengers [34], fewer head injuries (42 versus 55 %; *p* = 0.0001) were noted in a study of the outcomes of 1153 critically injured blunt trauma patients [17]. In the current study, more than half of the traumatic injuries were motorcycle accidents, with very few motor vehicle accidents, an injury pattern very different from those reported in Western countries. In motorcycle accidents, patients are protected only by their helmets, unlike the protection provided by cars. However, in car accidents, obese occupants do not sit in the same position as non-obese occupants; thus, restraint systems including seatbelt and airbags are not optimized for occupants of all anthropometries [34].

Likewise, obese blunt trauma victims are more likely to suffer pulmonary contusion as well as pelvic, extremity, and rib fractures [18]. Some authors reported that severely injured obese blunt trauma patients had more chest injuries and lower extremity fractures [17]. In this study, analysis of AIS revealed that the obese patients had sustained significantly higher rates of thoracic injury; however, they also had a lower incidence of pneumothorax (OR = 0.4, 95 % CI = 0.20–0.93; *p* = 0.026) and clavicle fractures (OR = 0.7, 95 % CI = 0.49–0.86; *p* = 0.003) than normal-weight patients. It is likely that a “cushion effect” protects obese patients from crash injuries to the thorax that would typically induce pneumothorax; similarly, the shape of the obese bodies may prevent direct contusion forces on the clavicle. However, further research is required to validate this observation.

In addition, the controversial effect of obesity on the incidence and site of fractures in the extremities of trauma patients had been reported [32]. In this study, obese patients presented a 2.1-fold greater incidence of humeral fractures but no significant difference in pelvic, tibial, or fibular fractures compared to those of normal-weight patients. Although there is a positive correlation between bone mineral density (BMD) and BMI [35], increased body fat has a negative effect on attaining peak bone mass and bone mineral content [36]. Increased weight is associated with a 1.7-fold increased fracture risk in obese boys, particularly for fractures incurred from low-energy mechanisms [37]. Obese patients involved in motor vehicle crashes are significantly more likely to have more severe distal femur fractures compared with non-obese patients [38]. Subjects with higher BMI are at higher risk of fractures in the humerus, lower leg, and ankle [32]. Furthermore, hip fractures in elderly obese individuals are significantly less common than among non-obese men [39]; however, these data are in contrast to a report of increased incidence of hip fracture after adjusting for BMD [40].

In the current study, no significant differences were found between obese and normal-weight patients regarding ISS, regardless of the subgroup of injury severity; NISS; TRISS; mortality; the proportion of patients admitted to the ICU; and LOS in the ICU. However, obese patients had a significantly longer hospital LOS than normal-weight patients, although the difference was not large. Notably, the hospital LOS was significantly longer among obese patients with pelvic, tibial, and fibular fractures than among normal-weight patients. This finding is consistent with previous reports that obesity increases the risk of non-union of fractures and complicates trauma recovery [20] and that delay of recovery is directly correlated with the severity of obesity [41]. In addition to the high risk of a range of medical conditions associated with obesity, including HTN, DM, cardiac disease, and pulmonary thromboembolism, their presence may also delay recovery [42]. Increased complications after surgical treatment of pelvic ring injuries [11] or hip fracture [43] may increase hospital LOS. Moreover, even medically stable obese patients are almost twice as likely to experience delayed fracture fixation due to surgeon preference [12]. Therefore, our finding that obese patients with a pelvic and lower leg fractures had significantly longer hospital LOS than normal-weight patients was not surprising.

Obesity has significant implications for not only the demographics and patterns of injury but also for the assessment, management, and outcomes of trauma. In this study, sex-based difference (male predominance), pre-existing comorbidities and chronic diseases (DM, HTN, and CHF), BAC (lower alcohol concentration), and different associated injures (fewer cranial, maxillary, mandibular, clavicle, and pneumothorax fractures as well as more humoral fractures) making the comparison a complex problem in determining the effects of obesity on outcomes in obese patients. It is also difficult to interpret the reported body of literature on the interaction between obesity and trauma, which vary widely in patient selection, stratification, and outcome definitions. The patterns of injury and outcomes for obese trauma patients may be distinct for different mechanisms of injury [44]. The effect of obesity on outcomes of traumatic injury remains inconclusive without a matched cohort prospective study. Therefore, the retrospective design with its inherent selection bias results in a major limitation of the current study. In addition, the lack of data regarding the circumstances of the mechanism of injury such as impact type and force and the use of any other protective materials also limit interpretation of the analyzed data. Moreover, patients declared dead on hospital arrival or at the accident scene were not included in the Trauma Registry Database, which potentially biases the assessment of mortality. Finally, the descriptive study design prevents assessment of the effects of any particular treatment intervention, and could only rely on the assumption of uniform assessment and management of obese and normal-weight patient populations.

## Conclusion

Obese patients had different injury characteristics, bodily injury patterns, and longer LOS in hospital, particularly those with pelvic, tibial, and fibular fractures, compared to normal-weight patients. However, this study failed to identify any relationship between obesity and injury severity, mortality, proportion admitted to the ICU, or LOS in the ICU.

### Availability od data and materials

The raw data in excel file under de-identification policy could be provided via the email of corresponding author upon request for research purpose only.

## Additional file

Additional file 1:
**Table S1:** The incidence of associated injuries in obese and normal-weight adult patients. **Table S2:** The length of stay in hospital for obese and normal-weight adult patients. (DOCX 20 kb)

## References

[1] Finucane MM, Stevens GA, Cowan MJ, Danaei G, Lin JK, Paciorek CJ, Singh GM, Gutierrez HR, Lu Y, Bahalim AN (2011). National, regional, and global trends in body-mass index since 1980: systematic analysis of health examination surveys and epidemiological studies with 960 country-years and 9.1 million participants. Lancet.

[2] Stevens GA, Singh GM, Lu Y, Danaei G, Lin JK, Finucane MM, Bahalim AN, McIntire RK, Gutierrez HR, Cowan M (2012). National, regional, and global trends in adult overweight and obesity prevalences. Popul health metrics.

[3] Ogden CL, Carroll MD, Kit BK, Flegal KM: Prevalence of obesity among adults: United States, 2011-2012. NCHS Data Brief. 2013;(131):1-8. http://www.cdc.gov/nchs/data/databriefs/db131.pdf. 24152742

[4] Chiou ST. 2014 Health Promotion Administration Annual Report. Health Promotion Administration. Taiwan: Ministry of Health and Welfare; 2015.

[5] OECD (2010). Obesity and the economics of prevention: fit not fat.

[6] Byard RW, Langlois NE (2011). Letter to the editor--Increasing body weight of motorcycle riders. J Forensic Sci.

[7] Neville AL, Brown CV, Weng J, Demetriades D, Velmahos GC (2004). Obesity is an independent risk factor of mortality in severely injured blunt trauma patients. Arch Surg.

[8] Andruszkow H, Veh J, Mommsen P, Zeckey C, Hildebrand F, Frink M (2013). Impact of the body mass on complications and outcome in multiple trauma patients: what does the weight weigh?. Mediators Inflamm.

[9] Liu T, Chen JJ, Bai XJ, Zheng GS, Gao W (2013). The effect of obesity on outcomes in trauma patients: a meta-analysis. Injury.

[10] Mock CN, Grossman DC, Kaufman RP, Mack CD, Rivara FP (2002). The relationship between body weight and risk of death and serious injury in motor vehicle crashes. Accid Anal Prev.

[11] Sems SA, Johnson M, Cole PA, Byrd CT, Templeman DC (2010). Elevated body mass index increases early complications of surgical treatment of pelvic ring injuries. J Orthop Trauma.

[12] Childs BR, Nahm NJ, Dolenc AJ, Vallier HA (2015). Obesity is associated with more complications and longer hospital stays after orthopaedic trauma. J Orthop Trauma.

[13] Ciesla DJ, Moore EE, Johnson JL, Burch JM, Cothren CC, Sauaia A (2006). Obesity increases risk of organ failure after severe trauma. J Am Coll Surg.

[14] Brown CV, Velmahos GC (2006). The consequences of obesity on trauma, emergency surgery, and surgical critical care. World J Emerg Surg.

[15] Hoffmann M, Lefering R, Gruber-Rathmann M, Rueger JM, Lehmann W (2012). The impact of BMI on polytrauma outcome. Injury.

[16] Alban RF, Lyass S, Margulies DR, Shabot MM (2006). Obesity does not affect mortality after trauma. Am Surg.

[17] Brown CV, Neville AL, Rhee P, Salim A, Velmahos GC, Demetriades D (2005). The impact of obesity on the outcomes of 1,153 critically injured blunt trauma patients. J Trauma.

[18] Boulanger BR, Milzman D, Mitchell K, Rodriguez A (1992). Body habitus as a predictor of injury pattern after blunt trauma. J Trauma.

[19] Diaz JJ, Norris PR, Collier BR, Berkes MB, Ozdas A, May AK, Miller RS, Morris JA (2009). Morbid obesity is not a risk factor for mortality in critically ill trauma patients. J Trauma.

[20] Akinnusi ME, Pineda LA, El Solh AA (2008). Effect of obesity on intensive care morbidity and mortality: a meta-analysis. Crit Care Med.

[21] Ferrada P, Anand RJ, Malhotra A, Aboutanos M (2014). Obesity does not increase mortality after emergency surgery. J Obes.

[22] Majdan M, Brazinova A, Wilbacher I, Rusnak M, Mauritz W (2015). The impact of body mass index on severity, patterns and outcomes after traumatic brain injuries caused by low level falls. Eur J Trauma Emerg Surg.

[23] Liu HT, Liang CC, Rau CS, Hsu SY, Hsieh CH (2015). Alcohol-related hospitalizations of adult motorcycle riders. World J Emerg Surg.

[24] Liu HT, Rau CS, Liang CC, Wu SC, Hsu SY, Hsieh HY, Hsieh CH (2015). Bicycle-related hospitalizations at a Taiwanese level I Trauma Center. BMC Public Health.

[25] Rau CS, Lin TS, Wu SC, Yang JC, Hsu SY, Cho TY, Hsieh CH (2014). Geriatric hospitalizations in fall-related injuries. Scand J Trauma Resusc Emerg Med.

[26] Jou RC, Yeh TH, Chen RS (2012). Risk factors in motorcyclist fatalities in Taiwan. Traffic Inj Prev.

[27] Physical status: the use and interpretation of anthropometry. Report of a WHO Expert Committee. World Health Organ Tech Rep Ser 1995, 854:1-452. 8594834

[28] Obesity: preventing and managing the global epidemic. Report of a WHO consultation. World Health Organ Tech Rep Ser 2000, 894:i-xii, 1-253. 11234459

[29] Tsai J, Ford ES, Zhao G, Li C, Greenlund KJ, Croft JB (2012). Co-occurrence of obesity and patterns of alcohol use associated with elevated serum hepatic enzymes in US adults. J Behav Med.

[30] Ely M, Hardy R, Longford NT, Wadsworth ME (1999). Gender differences in the relationship between alcohol consumption and drink problems are largely accounted for by body water. Alcohol Alcohol.

[31] Sifri ZC, Kim H, Lavery R, Mohr A, Livingston DH (2008). The impact of obesity on the outcome of emergency intubation in trauma patients. J Trauma.

[32] Premaor MO, Comim FV, Compston JE (2014). Obesity and fractures. Arq Bras Endocrinol Metabol.

[33] Brown CV, Rhee P, Neville AL, Sangthong B, Salim A, Demetriades D (2006). Obesity and traumatic brain injury. J Trauma.

[34] Tagliaferri F, Compagnone C, Yoganandan N, Gennarelli TA (2009). Traumatic brain injury after frontal crashes: relationship with body mass index. J Trauma.

[35] Zhao LJ, Jiang H, Papasian CJ, Maulik D, Drees B, Hamilton J, Deng HW (2008). Correlation of obesity and osteoporosis: effect of fat mass on the determination of osteoporosis. J Bone Miner Res.

[36] Weiler HA, Janzen L, Green K, Grabowski J, Seshia MM, Yuen KC (2000). Percent body fat and bone mass in healthy Canadian females 10 to 19 years of age. Bone.

[37] Davidson PL, Goulding A, Chalmers DJ (2003). Biomechanical analysis of arm fracture in obese boys. J Paediatr Child Health.

[38] Maheshwari R, Mack CD, Kaufman RP, Francis DO, Bulger EM, Nork SE, Henley MB (2009). Severity of injury and outcomes among obese trauma patients with fractures of the femur and tibia: a crash injury research and engineering network study. J Orthop Trauma.

[39] Premaor MO, Compston JE, Fina Aviles F, Pages-Castella A, Nogues X, Diez-Perez A, Prieto-Alhambra D (2013). The association between fracture site and obesity in men: a population-based cohort study. J Bone Miner Res.

[40] Nielson CM, Marshall LM, Adams AL, LeBlanc ES, Cawthon PM, Ensrud K, Stefanick ML, Barrett-Connor E, Orwoll ES (2011). BMI and fracture risk in older men: the osteoporotic fractures in men study (MrOS). J Bone Miner Res.

[41] Dhungel V, Liao J, Raut H, Lilienthal MA, Garcia LJ, Born J, Choi KC (2015). Obesity delays functional recovery in trauma patients. J Surg Res.

[42] Rosenfeld HE, Tsokos M, Byard RW (2012). The association between body mass index and pulmonary thromboembolism in an autopsy population. J Forensic Sci.

[43] Belmont PJ, Garcia EJ, Romano D, Bader JO, Nelson KJ, Schoenfeld AJ (2014). Risk factors for complications and in-hospital mortality following hip fractures: a study using the National Trauma Data Bank. Arch Orthop Trauma Surg.

[44] Osborne Z, Rowitz B, Moore H, Oliphant U, Butler J, Olson M, Aucar J (2014). Obesity in trauma: outcomes and disposition trends. Am J Surg.

