# Supplementary material for: Traumatic injuries among adult obese patients in southern Taiwan: a cross-sectional study based on a trauma registry system
Source: BMC Public Health. 2016 Mar 18;16:275. doi: 10.1186/s12889-016-2950-z (PMC4797357; doi:10.1186/s12889-016-2950-z)
Supplement: Additional file 1: — Table S1: The incidence of associated injuries in obese and normal-weight adult patients. Table S2: The length of stay in hospital for obese and normal-weight adult patients. (DOCX 20 kb) [file 12889_2016_2950_MOESM1_ESM.docx]

**Supplementary Table 1**

| Variables | Obese  BMI≥30  n=880 | Normal  25>BMI≥18.5  n=5391 | *Odds ratio*  *(95%)* | *P* |
| --- | --- | --- | --- | --- |
| Head trauma, n(%) |  |  |  |  |
| Neurologic deficit | 4(0.5) | 45(0.8) | 0.5(0.20-1.51) | 0.235 |
| Cranial fracture | 38(4.3) | 339(6.3) | 0.7(0.48-0.95) | 0.023 |
| Epidural hematoma (EDH) | 25(2.8) | 218(4.0) | 0.7(0.46-1.06) | 0.086 |
| Subdural hematoma (SDH) | 66(7.5) | 432(8.0) | 0.9(0.71-1.22) | 0.602 |
| Subarachnoid hemorrhage (SAH) | 65(7.4) | 455(8.4) | 0.9(0.66-1.13) | 0.293 |
| Intracerebral hematoma (ICH) | 10(1.1) | 103(1.9) | 0.6(0.31-1.13) | 0.109 |
| Cerebral contusion | 35(4.0) | 274(5.1) | 0.8(0.54-1.11) | 0.160 |
| Cervical vertebral fracture | 9(1.0) | 46(0.9) | 1.2(0.59-2.46) | 0.617 |
| Maxillofacial trauma, n(%) |  |  |  |  |
| Orbital fracture | 17(1.9) | 127(2.4) | 0.8(0.49-1.36) | 0.436 |
| Nasal fracture | 9(1.0) | 69(1.3) | 0.8(0.40-1.60) | 0.523 |
| Maxillary fracture | 36(4.1) | 407(7.5) | 0.5(0.37-0.74) | <0.001 |
| Mandibular fracture | 14(1.6) | 151(2.8) | 0.6(0.32-0.98) | 0.038 |
| Thoracic trauma, n(%) |  |  |  |  |
| Rib fracture | 88(10.0) | 446(8.3) | 1.2(0.97-1.57) | 0.089 |
| Sternal fracture | 1(0.1) | 9(0.2) | 0.7(0.09-5.38) | 1.000 |
| Hemothorax | 16(1.8) | 81(1.5) | 1.2(0.71-2.09) | 0.482 |
| Pneumothorax | 7(0.8) | 99(1.8) | 0.4(0.20-0.93) | 0.026 |
| Hemopneumothorax | 14(1.6) | 76(1.4) | 1.1(0.64-2.01) | 0.675 |
| Lung contusion | 9(1.0) | 49(0.9) | 1.1(0.55-2.30) | 0.744 |
| Thoracic vertebral fracture | 11(1.3) | 47(0.9) | 1.4(0.74-2.79) | 0.277 |
| Abdominal trauma, n(%) |  |  |  |  |
| Intra-abdominal injury | 15(1.7) | 85(1.6) | 1.1(0.62-1.88) | 0.779 |
| Hepatic injury | 11(1.3) | 112(2.1) | 0.6(0.32-1.11) | 0.101 |
| Splenic injury | 11(1.3) | 54(1.0) | 1.3(0.65-2.40) | 0.500 |
| Retroperitoneal injury | 1(0.1) | 9(0.2) | 0.7(0.09-5.38) | 1.000 |
| Renal injury | 7(0.8) | 27(0.5) | 1.6(0.69-3.67) | 0.316 |
| Lumbar vertebral fracture | 16(1.8) | 93(1.7) | 1.1(0.62-1.80) | 0.845 |
| Sacral vertebral fracture | 7(0.8) | 31(0.6) | 1.4(0.61-3.16) | 0.435 |
| Extremity trauma, n(%) |  |  |  |  |
| Scapular fracture | 15(1.7) | 96(1.8) | 1.0(0.55-1.66) | 0.874 |
| Clavicle fracture | 57(6.5) | 519(9.6) | 0.7(0.49-0.86) | 0.003 |
| Humeral fracture | 72(8.2) | 216(4.0) | 2.1(1.62-2.82) | <0.001 |
| Radial fracture | 96(10.9) | 577(10.7) | 1.0(0.81-1.28) | 0.855 |
| Ulnar fracture | 42(4.8) | 282(5.2) | 0.9(0.65-1.27) | 0.569 |
| Metacarpal fracture | 36(4.1) | 162(3.0) | 1.4(0.95-1.99) | 0.088 |
| Pelvic fracture | 34(3.9) | 158(2.9) | 1.3(0.91-1.94) | 0.136 |
| Femoral fracture | 85(9.7) | 471(8.7) | 1.1(0.88-1.42) | 0.372 |
| Patella fracture | 26(3.0) | 149(2.8) | 1.1(0.70-1.64) | 0.750 |
| Tibial fracture | 74(8.4) | 409(7.6) | 1.1(0.86-1.45) | 0.396 |
| Fibular fracture | 38(4.3) | 218(4.0) | 1.1(0.75-1.52) | 0.703 |
| Calcaneal fracture | 49(5.6) | 265(4.9) | 1.1(0.83-1.56) | 0.411 |
| Metatarsal fracture | 30(3.4) | 189(3.5) | 1.0(0.66-1.44) | 0.885 |

**Supplementary Table 2**

| Variables | Obese  BMI≥30  n=880 | Normal  25>BMI≥18.5  n=5391 | *P* |
| --- | --- | --- | --- |
| LOS |  |  |  |
| Head trauma, n(%) |  |  |  |
| Neurologic deficit | 9.3±4.3 | 13.1±10.3 | 0.462 |
| Cranial fracture | 14.5±9.4 | 13.1±12.3 | 0.489 |
| Epidural hematoma (EDH) | 17.4±10.4 | 15.5±14.3 | 0.538 |
| Subdural hematoma (SDH) | 12.9±11.8 | 15.5±16.8 | 0.215 |
| Subarachnoid hemorrhage (SAH) | 12.6±11.5 | 13.2±12.7 | 0.745 |
| Intracerebral hematoma (ICH) | 16.2±13.8 | 17.0±15.2 | 0.867 |
| Cerebral contusion | 10.8±8.7 | 16.5±14.9 | 0.002 |
| Cervical vertebral fracture | 9.6±15.6 | 16.8±15.8 | 0.212 |
| Maxillofacial trauma, n(%) |  |  |  |
| Orbital fracture | 7.4±6.2 | 11.7±7.9 | 0.033 |
| Nasal fracture | 15.1±8.7 | 10.4±6.7 | 0.059 |
| Maxillary fracture | 10.1±6.5 | 10.8±8.4 | 0.595 |
| Mandibular fracture | 12.8±7.6 | 12.2±9.4 | 0.818 |
| Thoracic trauma, n(%) |  |  |  |
| Rib fracture | 12.2±11.9 | 11.2±9.9 | 0.481 |
| Sternal fracture | 5.0±0.0 | 14.3±14.4 | — |
| Hemothorax | 17.3±13.8 | 14.8±12.1 | 0.466 |
| Pneumothorax | 11.0±5.1 | 15.9±13.8 | 0.354 |
| Hemopneumothorax | 19.7±9.5 | 17.2±13.3 | 0.503 |
| Lung contusion | 16.8±12.7 | 13.3±8.0 | 0.446 |
| Thoracic vertebral fracture | 18.3±23.3 | 18.7±16.3 | 0.945 |
| Abdominal trauma, n(%) |  |  |  |
| Intra-abdominal injury | 13.0±10.4 | 12.1±8.8 | 0.722 |
| Hepatic injury | 21.5±11.7 | 15.9±11.8 | 0.129 |
| Splenic injury | 17.2±12.5 | 15.5±12.3 | 0.675 |
| Retroperitoneal injury | 1.0±0.0 | 16.7±9.2 | — |
| Renal injury | 12.6±6.1 | 10.5±7.0 | 0.483 |
| Urinary bladder injury | — | — | — |
| Lumbar vertebral fracture | 24.0±20.5 | 13.6±11.5 | 0.066 |
| Sacral vertebral fracture | 23.4±19.4 | 17.7±12.1 | 0.479 |
| Extremity trauma, n(%) |  |  |  |
| Scapular fracture | 9.1±6.3 | 10.6±9.7 | 0.576 |
| Clavicle fracture | 8.9±8.1 | 8.6±9.7 | 0.820 |
| Humeral fracture | 10.0±11.0 | 8.9±9.0 | 0.411 |
| Radial fracture | 7.6±8.0 | 7.1±9.1 | 0.585 |
| Ulnar fracture | 9.5±9.9 | 8.7±10.3 | 0.640 |
| Metacarpal fracture | 11.2±11.8 | 7.7±7.6 | 0.100 |
| Pelvic fracture | 25.8±18.1 | 17.0±12.7 | 0.010 |
| Femoral fracture | 13.2±10.1 | 11.9±11.1 | 0.343 |
| Patella fracture | 9.3±7.1 | 8.7±8.2 | 0.726 |
| Tibial fracture | 19.8±16.4 | 13.6±12.1 | 0.002 |
| Fibular fracture | 18.3±12.9 | 13.6±11.9 | 0.027 |
| Calcaneal fracture | 12.7±10.0 | 10.1±9.7 | 0.092 |
| Metatarsal fracture | 13.9±11.7 | 12.3±12.1 | 0.498 |
